# Needle, Holder and Ligature in One Instrument

**Published:** 1919-11-22

**Authors:** 


### A NEW SUTURE METHOD.

### Needle, Holder and Ligature in one Instrument.

An interesting and valuable method of suture is described by Alfred Kahn, M.D., in the American *Medical Record* of September 6, and we draw attention to it as one which has great possible utility in closing operative or traumatic wounds in many of the generally considered inaccessible sites.

The idea centres chiefly in the instrument or needle itself, which is designed to do away with the threading and re-threading of needles as well as the always inconvenient needle-holder. The apparatus consists of a long hollow needle with a small hole near its sharp extremity. The other end is screwed to a hollow metal handle, inside which lies rolled a continuous thread. The thread is pulled from the roll through the centre of the instrument throughout its full length, leaving the instrument at the small hole in the needle and continuing thence far enough for use as a ligature.

### The Technique.

The technique is thus described. "The parts of the instrument having been joined together, both sides of the wound are pierced and the ligature pulled through. The instrument then retraces itself, and the ligature is cut away from the instrument and is tied. When the ligature is cut away there is enough of the needle left extending from the instrument through the hole to allow the next ligature to be unwound from the spool and the next ligature is ready. This process is repeated until the wound is entirely united."

### A Simple and Good Idea.

Considering this procedure critically there seems to be very little against it except some possible difficulty in sterilising the whole efficiently and keeping it so. In favour of it, we note that the firm attachment of the long needle to a satisfactorily large handle and the complete absence of needle-threading may be of great advantage in necessarily rapid and inaccessible operations. Also for fine, accurate suture work it may have considerable use. We have not ourselves had the opportunity of testing the actual working of this small machine, but in view of the complications and difficulties so often encountered in small, deep areas when small needles and long holders must be used, and particularly on occasions when quick and efficient assistants are lacking, we think that Dr. Kahn's good but simple idea is well worth the attention of those surgeons who are progressively inclined.

